# Nuclear miRNAs: Gene Regulation Activities

**DOI:** 10.3390/ijms25116066

**Published:** 2024-05-31

**Authors:** Monia Billi, Elisabetta De Marinis, Martina Gentile, Clara Nervi, Francesco Grignani

**Affiliations:** 1 General Pathology and Department of Medicine, University of Perugia, 06132 Perugia, Italy; billimonia@gmail.com; 2 Department of Medical-Surgical Sciences and Biotechnologies, University of Rome “La Sapienza”, 04100 Latina, Italy; elisabetta.demarinis@uniroma1.it (E.D.M.); martina.gentile@uniroma1.it (M.G.); clara.nervi@uniroma1.it (C.N.)

**Keywords:** miRNAs, nuclear localization, gene regulation, transcriptional control, RNA processing, hematopoiesis

## Abstract

MicroRNAs (miRNAs) are small non-coding RNAs which contribute to the regulation of many physiological and pathological processes. Conventionally, miRNAs perform their activity in the cytoplasm where they regulate gene expression by interacting in a sequence-specific manner with mature messenger RNAs. Recent studies point to the presence of mature miRNAs in the nucleus. This review summarizes current findings regarding the molecular activities of nuclear miRNAs. These molecules can regulate gene expression at the transcriptional level by directly binding DNA on the promoter or the enhancer of regulated genes. miRNAs recruit different protein complexes to these regions, resulting in activation or repression of transcription, through a number of molecular mechanisms. Hematopoiesis is presented as a paradigmatic biological process whereby nuclear miRNAs possess a relevant regulatory role. Nuclear miRNAs can influence gene expression by affecting nuclear mRNA processing and by regulating pri-miRNA maturation, thus impacting the biogenesis of miRNAs themselves. Overall, nuclear miRNAs are biologically active molecules that can be critical for the fine tuning of gene expression and deserve further studies in a number of physiological and pathological conditions.

## 1. Introduction

MicroRNAs (miRNAs) are classically regarded as negative regulators of messenger RNA (mRNA) function. This activity is mainly obtained through both the repression of mRNA translation and induction of mRNA degradation upon the binding of miRNAs to the 3′ untranslated region (3′ UTR) of mRNAs [1,2]. These functions of miRNAs take place within cellular cytoplasm, through their binding to mature mRNAs [3]. However, a number of reports indicate that miRNAs may possess wider activities that include the direct regulation of chromatin structure and transcription [4,5]. This type of regulatory activity could be activating or repressing, depending on the protein complexes that miRNAs drive to DNA and chromatin. Therefore, miRNAs are active within the nucleus where they must either remain or be transported back across the nuclear membrane.

This review briefly summarizes current evidence indicating such non-canonical nuclear activities of miRNAs, highlighting their biological roles in the regulation of hematopoiesis.

## 2. Biogenesis of MicroRNAs

**Figure 1:**
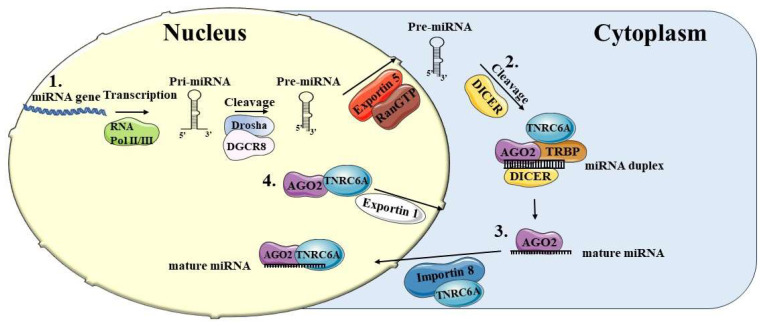
Biogenesis of miRNAs and their return to the nucleus. The biogenesis of miRNAs is mediated by several steps. (1) miRNA genes are transcribed by RNA Pol-II or Pol-III into primary miRNAs (pri-miRNAs) and then cleaved by Drosha and DGCR8 into precursor miRNAs (pre-miRNAs). They are then exported into the cytoplasm with the help of Exportin 5 and RanGTP. (2) The pre-miRNAs are cleaved into miRNA duplexes by DICER in the cytoplasm and associated with the Argonaute proteins (AGO), forming the mature miRNA. (3) Part of the mature miRNAs are translocated into the nucleus with the help of Importin 8 and TNRC6. (4) AGO2 and TNRC6 can be transported into the cytoplasm via Exportin 1 (also called XPO1 or chromosomal region maintenance 1 or CRM1). miRNA, microRNA; RNA Pol-II/III, RNA polymerase II/III; pri-miRNA, miRNA primary transcript; pre-miRNA, miRNA precursor; AGO2, Argonaute 2; TNRC6A, trinucleotide repeat containing 6; TRBP, transactivation response-RNA-binding protein.

The biogenesis of miRNAs takes place in the nucleus and cytoplasm in several steps (Figure 1). They are typically transcribed by RNA polymerase II or III as long primary miRNAs (pri-miRNAs) with an internal loop structure containing a double-stranded stem region and an apical loop. miRNAs are mainly transcribed by RNA Pol-II. However, a role for RNA Pol-III in this event has been also reported [6,7,8].

The pri-miRNAs are transformed into precursor miRNAs (pre-miRNAs), structures of approximately 70 nucleotides (nt), by means of a protein complex consisting of the RNase III enzyme Drosha and the DGCR8 protein (DiGeorge syndrome critical region 8 gene) that binds double-stranded RNA (Figure 1(1)) [9,10,11]. The pre-miRNAs are further processed after being translocated into the cytoplasm. The export mechanism involves a protein complex consisting of Exportin 5 (XPO5), a member of the karyopherin β family, and a GTPase (Ran GTP) [12,13] (Figure 1(1)). Pre-miRNAs in the cytoplasm are converted into 22 nt duplex miRNAs, including the passenger strand and the guide strand, through the cleavage of hairpin RNA by the enzyme DICER [14]. The ds-miRNA molecule is then loaded onto the miRNA-induced silencing complex (miRISC), a ribonucleoprotein complex consisting of the Argonaute (AGO1-4) and TNRC6A proteins. The AGO protein removes the passenger strand to form the mature miRNA molecule [15,16] (Figure 1(2)). The TRBP2 protein, which is part of the RISC loading complex, works together with DICER to contribute to the regulation of miRNA production [17], likely by increasing the stability of DICER-substrate complexes [18]. The AGO protein family contains several members. Among them, AGO2 performs the activity of cutting RNA molecules. AGO2 is important for miRNA maturation and for gene silencing mechanisms regulated by small RNAs [19,20]. Ohrt et al. [21] have found that miRISC is imported into the nucleus of mammalian cells and that the nuclear RISC (a complex of ~158 kDa) composed by AGO2 and miRNA is about 20-fold smaller than its cytoplasmic counterpart (a complex of nearly 3 MDa). Thereafter, other authors further characterized the composition of the nuclear RISC, demonstrating the presence of GW182/TNRC6 as well [22,23], thus constructing a complex of a higher molecular weight than previously reported.

In the study by Kalantari et al. [24], semi-quantitative mass spectrometry confirms that the association of AGO2 with GW182/TNRC6 and AGO3 is well conserved and more stable in both nucleus and cytoplasm [25]. In contrast, AGO2 interactions with DICER and TRBP are limited to the cytoplasm. The import process of miRNAs into the nucleus involves various nuclear transport receptor proteins [26,27]. It has been discovered that the nuclear and cytoplasmic movement of miRNAs is driven by Importin 8 and Exportin 1. These are two members of the Importin family that recognize the nuclear localization sequences in proteins and perform their active transport through the nuclear pore complex [28,29,30,31,32].

To enter the nucleus, miRNAs must first form a complex with the AGO protein, which binds to Importin 8 and TNRC6A [26,32] (Figure 1(3)). TNRC6A can move from the nucleus to the cytoplasm and back again as it possesses a nuclear localization signal (NLS) and a nuclear export signal (NES) [31,32]. Consequently, by interacting with TNRC6A, AGO2-miRNA complex enters into the nucleus. TNRC6A guides the miRNA-AGO complex to the target gene, in its promoter region complementary to the seed region. Thus, TNRC6A is a central protein involved in miRNA-mediated gene transcription regulation [33,34]. Furthermore, AGO-TNRC6 complex can move to the cytoplasm interacting with Exportin 1 (Figure 1(4)) [35]. In contrast, DICER and TRBP enter the nucleus without being linked to RISC.

## 3. Evidence of miRNAs in the Nucleus

Meister et al., by studying Hela cells, demonstrated that mature miR-21 can be found in the cytoplasm and in the nucleus [36]. This discovery suggested that a number of mature miRNAs may be transported back into the nucleus, where they may exert a functional activity [36,37]. In 2009, Földes-Papp et al. used femtosecond laser microscopy to assess the association between molecular beacon probes and human single-stranded cellular targets of miR-122 [38]. They demonstrated that mature miR-122 from the cytoplasm enters the nucleus of human hepatocytes. In recent years, more and more studies have highlighted the presence of mature nuclear miRNAs in most mammalian cells thanks to high-throughput analyses [39,40]. Several studies have studied the distribution of microRNA in the cytoplasm and nucleus, taking advantage of high performance next-generation sequencing and microarrays [40,41]. A sequencing study of nuclear and cytoplasmic fractions deriving from endothelial cells subjected to hypoxia highlighted an increased localization of miR-210-3p in the nucleus. By using confocal microscopy, we studied miR-223 localization during retinoic acid-induced granulopoiesis in HL60 cells and primary blasts. In these conditions, mature miR-223 translocates to the nucleus and shows an increasing nuclear pattern. Furthermore, in situ hybridization experiments showed that following treatment with retinoic acid, miR-223 shows the ability to bind metaphase chromosomes, where RNA transcription is absent or minimal [42]. The study of the sequence of nuclear miRNAs has highlighted the existence of a nuclear localization signal (AGUGUU) at their 3′ terminal sequence [39]. Some nuclear miRNAs such as miR-193b, miR-19, miR-30b, miR-30c, miR-590-5p, miR-374 and miR-374b, contain very similar nuclear localization motifs in their sequence with small variations, including UGUGUU, ACUGUU, AGAGUUU, AGUCUUU, AGUGAU, AGUGUA, AGNGUN [4,43]. It is hypothesized that these specific sequences targeting miRNAs to the nucleus are recognized by the nuclear pore structures, allowing their entry. Interestingly, the nuclear localization of a specific miRNA varies based on the tissue type. For example, miR-29b has a nuclear localization in HeLa cells but not in other cell lines [44], suggesting a tissue-specific functional role of nuclear localization. Overall, the significant number of recent studies showing the nuclear localization of miRNAs support their functional role into the nucleus.

## 4. Functions of miRNAs in the Nucleus

Many functions of nuclear miRNAs have been reported in recent years. Very important among them is their ability to bind complementary sequences located on gene promoters or enhancers. The action of nuclear miRNAs associates with mechanisms of epigenetic modifications of DNA or chromatin status at the targeted genomic sites, leading to transcriptional activation or repression of target genes [45]. Nuclear miRNA activity depends also on the genomic location of binding sequences and on the epigenetic status at these sites (such as presence of TATA box motifs or CpG island regions) [46]. In addition, miRNA target regions can be located far away from the transcription start site (TSS) [47].

Three main models were proposed for miRNA interaction with RNA and DNA in the nucleus. In all cases, these models require AGO proteins in association with nuclear miRNAs and need further studies to be validated in vivo [22].

(i) RNA-RNA model: miRNAs conjugated with AGO target non-coding transcripts and act as a molecular scaffold to recruit epigenetic regulators that eventually alter chromatin accessibility [48,49,50,51,52].
(ii) RNA-DNA hybrid: this model implies the interaction of the seed sequence of miRNAs, complexed with AGO, with complementary sequences of single-stranded DNA when the double helix unwinds in proximity of the site of transcription initiation. This interaction may alter the binding of transcription factors or the process of histone modifications [53,54,55,56].
(iii) RNA-DNA triplex: a triple helical structure is formed between pyrimidine-rich miRNAs and purine-rich duplex DNA via Hoogsteen or reverse Hoogsteen interaction in the major groove of dsDNA, altering the accessibility of transcription factors [57,58,59].

### 4.1. Transcriptional Activation

**Figure 2:**
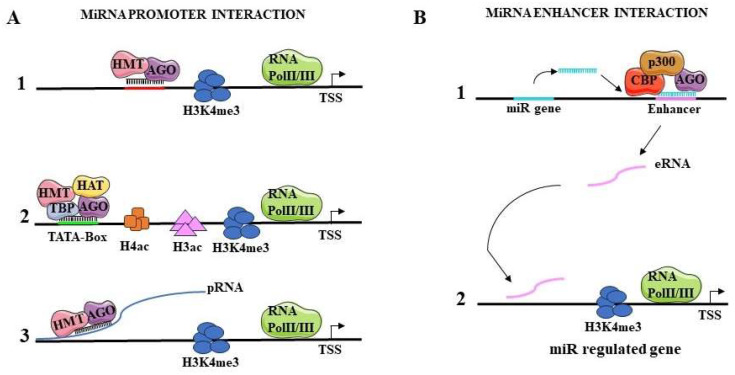
The activating function of nuclear miRNAs. (**A**) miRNA-mediated gene promoter regulation. (1) Direct interaction between miRNAs and complementary sequences on target gene promoters with the presence of AGO. This interaction allows an activator protein complex to be located in the vicinity of the targeted promoter region, whose chromatin structure is enriched with activator markers such as H3K4me3. RNA Pol-II is recruited and the gene is transcribed. (2) The miRNA-AGO-TBP complex interacts directly with the TATA box motif present on the promoter recruiting HMT and HAT. Chromatin is modified, activating markers such as H3K4me3, the H3ac and H4ac are increased, and RNA Pol-II is recruited to initiate transcription. (3) RNA Pol-II transcribes the promoter into promoter-associated RNA (pRNA). AGO mediates miRNA binding to pRNA, recruits HMT by producing active histone modifications, such as H3K4me3 with RNA polymerase II enrichment. (**B**) Transcription regulation through interaction with enhancers (1). A miRNA is transcribed from its gene located near enhancer loci. The mature miRNA forms a complex with AGO and p300/CBP, inducing active chromatin markers in the enhancer regions, and RNA Pol-II call. Thus, the enhancer is transcribed. (2) Next, the eRNA binds to p300 and other proteins to activate the target gene promoter. miRNAs, microRNAs; RNA Pol-II, RNA polymerase II; H3, histone H3; AGO, Argonaute 1 or Argonaute 2; TBP, TATA-Box-binding protein; eRNA, enhancer RNA; HAT, histone acetyltransferase; HMT, histone methyltransferase; pRNA, promoter-associated RNA.

Numerous studies show how miRNAs can mediate transcriptional gene activation (TGA) by interacting with the promoters (Figure 2A), or with enhancers (Figure 2B), of their target genes.

#### 4.1.1. Interaction with Promoters

One mechanism by which miRNAs activate gene transcription could be the association with promoters and their epigenetic regulation. miR-589 targets the promoter RNA of cyclooxygenase 2 (COX-2) by recruiting AGO2 and GW182 (TNRC6A) to form a complex. This complex can modify histones through association with WDR5, a protein that stimulates histone methyltransferase activity. Consequently, in the COX-2 promoter H3K4me3 and H4 acetylation levels increase, and gene transcription is activated [50] (Figure 2A(1)). FOXO3, an important gene in the regulation of ovarian follicular development and atresia, is regulated by miR-195-5p. This miRNA enters the cellular nucleus, associates with AGO2 and interacts with the FOXO3 promoter by recognizing a complementary sequence in the TATA box (Figure 2A(2)). Consequently, histone acetylation, hypomethylation, and transcriptional activation take place [60]. Transcriptional activation markers such as acetylated H3 and H4 and H3K4me2 are enriched in the promoter of the interleukin (IL) tumor suppressor genes IL24 and IL32, at specific sites bound by miR-205. As a consequence, RNA polymerase II (Pol-II) is recruited and, subsequently, transcribes the IL24 and IL32 genes [61,62]. miR-744 and miR-1186 increase transcription of the cyclin B1 gene (Ccnb1), by interacting with sequences on this gene promoter, which are highly complementary to their respective seed regions. Moreover, miR-744 association with AGO1 causes an enrichment of Pol-II and H3K4me3 marks at the Ccnb1 TSS [27].

Overall, three types of interactions have been hypothesized between miRNAs and the promoters of the genes that are regulated at the transcriptional level. One type of interaction (Figure 2A(1)) leads to the formation of a direct miRNA-DNA double helix complex. In this case, miRNA binds the promoter in association with AGO proteins and recruits histone modifiers that increase the levels of activating markers, such as H3K4me3, while decreasing the inhibitory histone marks [58]. In a second model, the miRNA-AGO complex interacts directly with the TATA box motif region or sites on the promoter also bound by transcription factors (Figure 2A(2)). This binding event leads to the recruitment of TATA box-binding protein (TBP), Pol-II and histone-modifying proteins such as methyltransferases or acetyltransferases, resulting in gene transcriptional activation [22,54]. Among miRNAs that act according to this model are miR-181d, let-7i and miR-138, which activate transcription of c-myc, IL-2 and insulin genes, respectively, by interacting with the TATA box on the promoter of these genes. In a last model, miRNAs bind to the promoter-associated sense or antisense RNA (pRNA) transcribed by the target gene promoter. Again, as in the other models, this interaction recruits transcription factors, histone modifiers and Pol-II to transcriptionally regulate gene expression [50,63,64] (Figure 2A(3)).

#### 4.1.2. Interaction with Enhancers

miRNA may mediate transcriptional activation through the regulation of enhancers. In this case, miRNA binding loci are located in the proximity of enhancer regions displaying the typical histone marks of functional enhancer regions, such as H3K27ac (Figure 2B). Subsequently, they form a complex with AGO2 and p300/CBP, which is able to increase the levels of H3K27ac and H3K4me1 and to decrease those of H3K27me3 on the enhancer region surrounding the miRNA locus. These histone modifications induce the interaction of Pol-II with the enhancer, the recruitment of transcription factors and the expression of enhancer RNA (eRNA) (Figure 2B(1)). Once transcribed, the eRNA interacts with the promoter of nearby genes to promote their transcription (Figure 2B(2)). The gene locus of miR-26a-1 is located near the genes coding for the ITGA9, CTDSPL, VILL and PLCD1 proteins [65,66]. It has been shown that when miR-26a-1 is overexpressed, there is a transcriptional activation of the ITGA9 and VILL genes. The seed sequence of miR-26a-1 and the enhancer sequence of these genes shows complementarity. When the miRNA seed or the enhancer sequence of the two genes is deleted or mutated, no transcriptional activation is obtained. Similarly, an enrichment of H3K27ac, a marker of enhancer regions, is detectable near the genomic locus of miR-3179, inducing transcriptional activation of adjacent PKD1P and ABCC6 genes [67]. In breast cancer cells, Liang et al. observed that miR-339 interacts with the enhancer of the neighboring gene GPER resulting in its upregulation [68]. A similar mechanism was observed for miR-24-1, that interacts with the enhancers of the neighboring genes FBP1 and FANCC. In association with AGO2, it promotes the transcription of these genes [67]. In this model, the transcribed eRNA is ready to interact with specific sites in the promoters of the target genes. Using microarrays, several enhancers were identified that exhibit sequence complementarity with the seed region of miR-24-1. The ectopic overexpression of miR-24-1 activates distant target genes, such as the KDM6B gene, by increasing the transcription of their enhancers [67]. Thus, miRNA-induced transcriptional activation of target genes via enhancer regulation can affect the expression of genes residing on the same genomic locus as well as distantly located genes.

### 4.2. Transcriptional Repression

**Figure 3:**
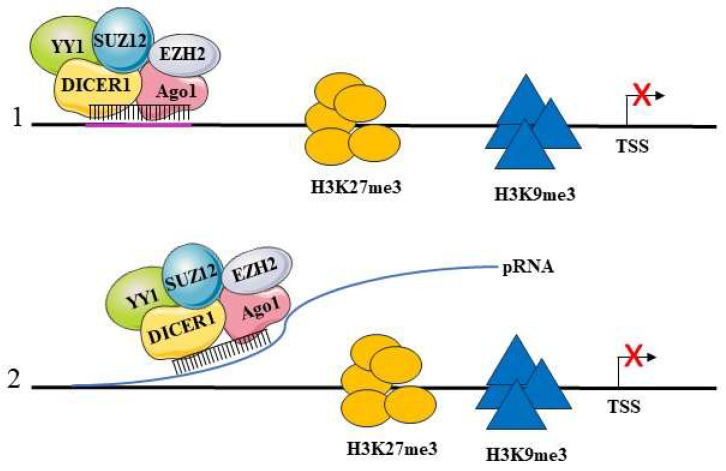
The suppressive function of nuclear miRNAs. miRNAs can inhibit gene expression at transcriptional level. The miRNA-AGO1 complex recruits a protein complex consisting of YY1, DICER1, SUZ12, EZH2 that promotes inhibitory chromatin modifications with increased H3K27me3 and H3K9me3 markers. This can occur through direct binding to the promoter via sites complementary to the seed region (1) or through binding to the pRNA, a promoter associated non-coding transcript (2). In this way, miRNA leads to a decrease in RNA Pol-II and the suppression of target genes. YY1, Yin Yang 1 transcription factor; EZH2, Enhancer of Zeste Homolog 2; SUZ12, Suppressor of Zeste 12 protein homolog; TSS, transcription start site, pRNA, a promoter-associated RNA.

Many studies show that mature nuclear miRNAs can repress the transcription of their target genes. Among the known mechanisms that are used for this regulation is their direct interaction with the promoter through the recognition of DNA sequences highly complementary with the miRNAs seed region. We have shown that this mechanism is used by miR-223 to regulate NFI-A gene transcription during granulopoiesis. We showed that miR-223 localizes in the nucleus and binds to two evolutionarily conserved regions in the NFI-A gene promoter, which are complementary to the miR-223 seed sequence. miR-223 recruits to that genomic region a protein complex consisting of AGO1, DICER1 and Polycomb proteins (PcG) YY1 and SUZ12, which are responsible for the trimethylation of H3K27 (H3K27me3) [69,70]. Thus, miR-223 generates an inactive chromatin state on the NFI-A gene promoter, which in turn blocks its expression. This event is required to direct the granulocytic differentiation of hematopoietic progenitors [42]. Benhamed et al. demonstrated that nuclear miRNAs of the let-7 family, in particular by let-7f, acts on the promoters of genes involved in cellular senescence and induces their transcriptional gene silencing [71]. CDC2 and CDCA8 are genes of the RB1/E2F complex and are repressed during cellular senescence. The sequence of their promoters shows sites complementary to let-7f, where the association of miRNA with a protein complex consisting of AGO2, RB1 and E2F occurs. This results in the recruitment at these sites of histone methyltransferases (HMTs) and histone deacetylases (HDACs), which induce an increase in repressive H3K27me3 and H3K9me2 histone marks, paralleling a decrease in the activating H3K4me3 marks and leading to a repression of the expression of genes involved in cellular senescence. Our unpublished data on acute promyelocytic leukemia cells induced into myeloid differentiation by treatment with retinoic acid show an increased expression of let-7c, which upon this treatment translocates into the nucleus of differentiated cells. In addition, whole-genome chromatin IP sequencing identifies a number of genes that are bound by let-7c and are relevant for myeloid differentiation. These genes can be transcriptionally activated or repressed, following an accumulation in their chromatin of activating H3K4me3 or repressing H3K27me3 marks, respectively. These data further confirm that nuclear miRNAs can perform activatory and repressive transcriptional functions. In gastric cancer, miR-584-3p interacts with the promoter of MMP14 (matrix metalloproteinase 14), at an upstream region of the TSS, and represses its transcription. This regulation occurs as a result of the recruitment of a protein complex consisting of AGO2, EZH2 and EHMT2 to the promoter. This results in increased levels of H3K27me3 and H3K9me2 characteristic of inactive chromatin [53] (Figure 3(1)). Genes involved in cardiac hypertrophy are transcriptionally repressed by the interaction of miR-208b with the promoters and recruitment of EZH2. As a consequence of EZH2 binding, the chromatin of these promoters is enriched with inhibitory markers (H3K27me3) [72]. In addition, miR-320 interacts with the gene promoter of the RNA polymerase III subunit D (POLR3D), where it recruits the EZH2 and AGO1 proteins. This results in transcriptional silencing of the POLR3D gene following the accumulation of the inhibitory marker H3K27me3 [73]. A peculiar mechanism of repression is operated by miR-126-5p to support endothelial integrity and counteract atherosclerosis. miR-126-5p in a complex with AGO2 is transported to the nucleus by means of its association with Mex3a, an RNA binding protein. Within the nucleus, AGO2 is released from miR-126-5p, which then is found associated with caspase-3. The association blocks the activity of caspase-3 since it impairs its ability to dimerize, leading to apoptosis inhibition. Thus, in this mechanism, a nuclear miRNA is able to block the function of a protein through the formation of a riboprotein complex. Biologically, this leads to the protection of endothelial cells from apoptosis in conditions of shear stress during the process of atherosclerosis [74]. Some miRNAs appear to function as transcriptional and post-transcriptional regulators of the same target gene. As described above, this concept applies to let-7 family miRNAs [71] and to the regulation of the expression of the NFI-A gene during granulopoiesis by miR-223 [42] (see below). miR-552 also has a dual regulatory effect at both the transcriptional and post-transcriptional levels. This miRNA directly binds the CYP2E1 gene promoter, but this interaction occurs through a region of the miRNA that is not the seed region. At the same time, miR-552 performs a classical post-transcriptional regulation through the binding of the 3′ UTR of CYP2E1 mRNA [75]. As mentioned above, miRNAs may regulate transcription by interacting with RNA produced by promoter sequences. Some promoters are transcribed into an antisense strand, producing a pRNA, containing sequences complementary to miRNAs [76]. The miRNA interaction with the antisense pRNA transcript occurs through the seed sequence and results in transcriptional repression of the target gene. Following this binding, an inhibitory protein complex consisting of EZH2, YY1, AGO, DICER1 and SUZ12 is recruited to the promoter, resulting in chromatin modification and an increase in the repressor markers H3K27me3 and H3K9me2 (Figure 3(2)). An example of this regulatory pattern is the activity of miR-423-5p on the antisense transcript of the promoter of the gene coding for the progesterone receptor (PR). It has been observed that as a result of this interaction, miR-423-5p recruits AGO2 and histone modifiers that increase the level of histone H3K9me2 in the PR promoter leading to transcriptional silencing of the gene [77].

## 5. Strategies to Determine the Function of Nuclear microRNAs in Mammalian Cells

A significant number of studies have elucidated the functional roles of nuclear miRNAs in several cellular contexts, including differentiating cells, cancer cells and terminally differentiated cells, as reported above in Section 3 and Section 4. Diverse technical approaches have been used to elucidate the actual subcellular localization and function of miRNAs and the integration of different methodologies was crucial for obtaining meaningful data.

### 5.1. Identification of microRNAs in the Nucleus

The following approaches have all been utilized to identify miRNAs in the nucleus.

- *Subcellular fractionation coupled with qRT-PCR, ddPCR or miRNA-sequencing.* The nuclei-cytosol fractionation is a critical step to properly demonstrate a nuclear-localized miRNA, although not sufficient to properly support its activity. Commercially available kits for nuclei-cytosol isolation are optimized for commonly used cell lines, but their usage needs consistent adjustments to obtain good purity of fractions from primary cells [41,78]. A validation of nuclear fraction purification can be accomplished using the highly sensitive droplet digital PCR (ddPCR), which allows the detection and quantification of subcellular-specific RNAs (such as MALAT1 or RNU6 for the nuclear fraction, and TUG1 or 18S rRNA for the cytosolic fraction) [79]. Following ddPCR, qRT-PCR or miRNA sequencing (miRNA-seq) are used to identify and assess the expression of miRNA(s) in the nuclear fraction. In particular, miRNA-seq provides comprehensive profiling of miRNA expression in the nucleus.
- *In situ hybridization and fluorescent confocal microscopy.* These strategies enable the visualization of miRNA localization within the nucleus together with other RNAi machinery components. miRNA detection can be precisely accomplished using fluorescently labeled double-stranded oligonucleotides, mimicking endogenous mature miRNAs (mimic-miRNA) or labeled locked nucleic acids (LNA) probes. LNAs are modified nucleotides with a constrained conformation due to a methylene bridge connecting the 2′ oxygen and 4′ carbon atoms of the ribose sugar. This modification enhances the binding affinity and stability of LNA probes to their complementary miRNA targets, resulting in increased sensitivity and specificity in miRNA detection. LNA probes are commonly employed in in situ hybridization experiments to visualize and localize specific miRNA molecules within cell nuclei by fluorescent confocal microscopy [42,80,81,82].
- *DNA affinity precipitation.* This precipitation-based technique requires the transfection of 3′-end biotin labeled miRNA into cells, formaldehyde crosslinking, DNA shearing and a streptavidin-mediated pull-down of miRNA-DNA complexes. Subsequently, miRNA binding sequences can be screened by next-generation sequencing in the whole genomic regions [83].
- *RNA Immunoprecipitation (RIP).* This method involves the use of antibodies specifically recognizing different components of the RNAi machinery (e.g., AGO proteins or DICER) to immunoprecipitate miRNA-protein complexes from nuclear extracts [42,50].

### 5.2. MicroRNA Activity in the Nucleus

Once nuclear miRNAs are identified, the functional validation of miRNA activity within the nucleus is provided by their interactions with the genome and consequent modulation of gene expression. Several methods generated data in this respect.

- *Bioinformatics*. Several bioinformatic tools have been developed to predict genomic targets of miRNA within gene promoters or given sequences. These tools are set up with algorithms that integrate seed region matching, structure, evolutionary conservation, machine learning and thermodynamic stability [5,22,84]. Experimental validation steps are essential thereafter, since nuclear miRNA matching on genome may be non-seed region related [61,75] and may extend beyond gene promoters [61,78,83].
- *Reporter assays.* These assays involve recombinant plasmids including reporter genes (e.g., GFP, luciferase) under the control of the genomic regions potentially targeted by nuclear miRNA. Validation of the actual matching implicates overexpression or inhibition of the nuclear miRNA, as well as mutagenesis of either the miRNA seed region or the targeted DNA sequence [61,81,82,85]. Although several drawbacks due to non-physiological concentration of miRNA and/or its genomic target, the investigation can be implemented by examining the co-occurrence of chromatin modifiers or transcription factors on the miRNA-target site, thereby elucidating the functional significance of nuclear miRNA binding [84].
- *Chromatin Immunoprecipitation (ChIP) and ChIP-sequencing.* Immunoprecipitation of chromatin with AGO proteins, in conjunction with histone marks, RNA Pol-II, or chromatin modifiers, adds an extra layer of investigation to explore the functional role of nuclear miRNAs in transcriptional gene activation or silencing. Furthermore, ChIP-sequencing enables a comprehensive investigation at the genome-wide level, uncovering the overall mechanisms and pathways influenced by nuclear miRNAs and their genomic counterparts [27,42,61,85,86].

**Figure 4:**
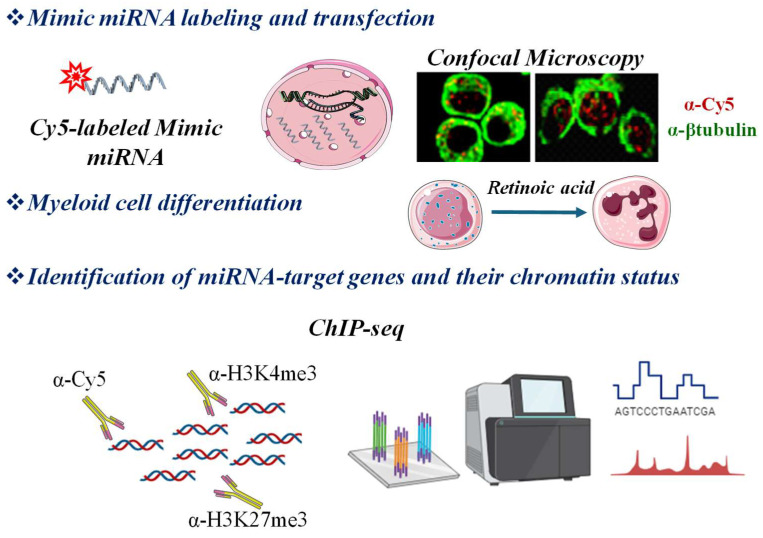
Graphical sketch of an experimental procedure used for the identification of nuclear miRNA genomic targets and epigenetic status at these sites. Transfection of fluorophore (i.e., Cy5)-labeled mimic miRNA allows for the visualization by confocal microscopy of its subcellular localization. In our model, Cy5-labeled mimic miR-223 (red signals) enters in the nucleus of myeloid cells after retinoic acid-induced differentiation. βtubulin (green signals) marks the cell cytosol [42]. Within this methodological approach, antibodies against Cy5 (α-Cy5) and against histone marks (α-H3K4me3 and α-H3K27me3) can be utilized to immunoprecipitate chromatin, enabling the identification across the whole genome through ChIP-sequencing of the complementary sequences targeted by nuclear miRNA and the epigenetic status at these sites. Portions of the figure were created with BioRender.com and Servier Medical Art, licensed under CC BY 4.0. For details, see text.

To precisely demonstrate the functional presence of an miRNA in the nucleus, our group used the transfection of Cy5-labeled double-stranded oligonucleotides (miR-Cy5), mimicking the activity of endogenous miR-223, to demonstrate by chromatin immunoprecipitation (ChIP) the enrichment of miR-223-Cy5 on a region of the NFI-A gene promoter, containing DNA sequences complementary to miR-223. ChIP assays using antibodies recognizing trimethylation of lysine 4 or 27 on histone H3 (H3K4me3, H3K27me3), were used to detect the chromatin status at these sites. Using confocal microscopy and RIP assays, we reported that miR-223 is part of a ribonucleoprotein repressive complex involving YY1, DICER1 and, transiently, AGO1 [42,87]. We also propose that this strategy can be extended to the whole genome using ChIP-sequencing (ChIP-seq). This would enable the identification of all genomic sites directly bound by fluorophore-labeled mimic miRNAs and concurrent enrichment of histone marks (Figure 4).

Nonetheless, technical challenges and biological questions still persist and future experimental strategies should consider both the context-dependent subcellular localization of miRNAs and their biological effects, while also distinguishing the specific role of a nuclear-localized miRNA from its cytosolic contribution [78,84].

## 6. Nuclear miRNAs in Hematopoiesis

Many studies have related the differential expression and activity of miRNAs with the regulation of hematopoietic differentiation and leukemic transformation [88,89,90,91,92,93]. In particular, differentiation factors, cell cycle regulators or transcription factors that are involved in hemopoiesis appear to be targets of miRNAs. By studying the expression profiles of miRNAs at different stages of hematopoietic differentiation, some of the key regulators have been identified [94,95,96,97,98]. Among these miRNAs, some show a predominantly nuclear localization. However, there is limited work evaluating the nuclear role of these miRNAs and their involvement in hematopoietic differentiation. We showed that miR-223 performs a dual regulation on the NFI-A gene, by virtue of both its cytoplasmic and nuclear component. The cytoplasmic component has a major regulatory role in hematopoiesis. miR-223 transcription is repressed by NFI-A, whereas it is increased by the transcription factor CEBPα, which induces myeloid differentiation of granulocyte–monocyte progenitors. In turn, cytoplasmic miR-223 represses NFI-A translation [99]. In addition to these activities, as described above in this review, we demonstrated that miR-223 localizes in the nucleus of hematopoietic cells, where it transcriptionally represses NFI-A gene expression to promote granulocyte differentiation [42]. In fact, the levels of miR-223 are finely regulated during hematopoiesis [100]; the transcriptional repression of miR-223 expression by the oncogenic fusion protein AML1/ETO triggers the development of leukemia [100]. A study of the nuclear expression profiles of miRNAs during mouse granulopoiesis identified an accumulation of miR-690 in the nucleus. This miRNA targets the CEBPα gene; when miR-690 is overexpressed, CEBPα is downregulated. The retention of miR-690 in the nucleus decreases the share of cytoplasmic miR-690, with consequent increased expression of CEBPα and myeloid differentiation. miR-706 is also enriched in the nucleus of myeloid cells, with a decrease in its cytoplasmic localization in granulocytes compared to promyelocytes. This miRNA would appear to negatively regulate the STAT1 transcription factor, which is important for myeloid differentiation [101,102]. Thus, the accumulation of miR-706 in the nucleus would decrease its activity as a negative regulator of STAT1 in the cytoplasm, thus favoring myeloid differentiation. It has also been reported that miR-709 binds with perfect complementarity to pri-miR-15a and pri-miR-16-1 and inhibits the production of mature miRNAs [103]. Therefore, nuclear miRNAs can regulate the expression of other miRNAs through a novel interaction with their immature precursors. miR-709 increases its expression and nuclear localization in granulocytes compared to promyelocytes. Several studies also show that miR-709 post-transcriptionally represses c-myc, whose expression levels are downregulated during myeloid cell differentiation [41]. Overall, the analysis of miR-690, miR-706 and miR-709 reveals new important mechanisms of functioning in the process of granulopoiesis. A first mechanism is the interaction and blockade at the nuclear level of the primary transcripts of other miRNAs. This results in the repression of the miRNAs and increased expression of their target genes. The other rather novel and peculiar mechanism is that miRNAs can be retained in the nucleus to decrease their cytoplasmic levels. As a consequence, the expression of its cytoplasmic target genes is increased [41].

## 7. Non-Transcriptional Activities of Nuclear miRNAs

Nuclear miRNAs are reported to exert post-transcriptional regulation of RNA. Several studies show the interaction between miRNAs and mRNA in the nucleus, where miRNAs recognize and bind to miRNA’s responsive elements (MRE) sequences in the 3′ or 5′ UTR of the mRNA [104]. This interaction leads to the degradation of the mRNA, often involving AGO2 [105], but the mechanism of this effect has yet to be defined. miRNAs can also target long non-coding RNAs (lncRNAs) within the nucleus. For example, miR-210 is reported to interact with a specific sequence present on XIST [105], a long non-coding RNA involved in mammalian X-chromosome inactivation during early female embryogenesis. XIST is also associated with cancer. In particular, its inappropriate expression/localization on active X-chromosome is associated with breast cancer where miR-210 is highly expressed [82,106,107,108], further suggesting a regulatory activity of this miRNA on XIST. miRNAs also locate within the nucleolus, where they may exert their activity by regulating the pri-miRNA levels. In particular, miR-122 localizes in the nucleolus and binds to specific sequences on pri-miR-21. This interaction suppresses the miR-21 maturation induced by the Drosha complex and DGCR8 [109]. Other nucleolar miRNAs have been shown to interact with rRNAs. An example is miR-92a-2-3p, which recognizes and binds a site on the 28S rRNA [110]. Other authors show an effect on the biogenesis of rRNAs, since the suppression of a minimal RISC formed by AGO2 and miRNAs leads to an increase in the amount of ribosomal RNA, suggesting a repressor role of miRNAs on rRNAs [111,112]. Lastly, it has been observed that miRNAs interact with their target mRNA already in the nucleolus. Here, they exert an initial pre-repression on the mRNA that will be completed in the cytoplasm [113]. In conclusion, the data suggest that nuclear miRNAs may act in part by regulating RNA processing.

## 8. Conclusions and Future Perspectives

It is well established that miRNAs play a critical role in many biological processes such as cell differentiation, lineage specification, cell cycle and immune response. In the common view, the activity of miRNAs is mostly restricted to the cytoplasm, where they regulate mRNA stability and translation. Our review highlights current evidence showing that miRNAs possess alternative mechanisms of action that involve a nuclear activity. In the nucleus, miRNAs regulate gene expression through complex molecular mechanisms, including the regulation of transcription. miRNAs can promote both activation and inhibition of transcription of their target genes, which are defined by miRNA-complementary sequences in their genomic loci. Thus, alteration of the miRNA biogenesis mechanism and miRNA expression levels may influence biological processes at multiple levels, including gene transcription. Indeed, the complexity of chromatin remodelers and miRNA-associated machineries contributes to dynamically modify the modality of genomic interaction, according to cell type, cell differentiation programs and cell metabolic status, requiring further focused studies. This generates a wide range of pathological consequences, including cell degeneration and neoplastic transformation. In this context, the activity of nuclear miRNAs needs further investigation in both physiological and pathological settings, especially in the definition of their role in tumorigenesis.
